# Adaptation of Endothelial Cells to Physiologically-Modeled, Variable Shear Stress

**DOI:** 10.1371/journal.pone.0057004

**Published:** 2013-02-14

**Authors:** Joseph S. Uzarski, Edward W. Scott, Peter S. McFetridge

**Affiliations:** 1 J. Crayton Pruitt Family Department of Biomedical Engineering, University of Florida, Gainesville, Florida, United States of America; 2 Department of Molecular Genetics and Microbiology, University of Florida, Gainesville, Florida, United States of America; UAE University, Faculty of Medicine & Health Sciences, United Arab Emirates

## Abstract

Endothelial cell (EC) function is mediated by variable hemodynamic shear stress patterns at the vascular wall, where complex shear stress profiles directly correlate with blood flow conditions that vary temporally based on metabolic demand. The interactions of these more complex and variable shear fields with EC have not been represented in hemodynamic flow models. We hypothesized that EC exposed to pulsatile shear stress that changes in magnitude and duration, modeled directly from real-time physiological variations in heart rate, would elicit phenotypic changes as relevant to their critical roles in thrombosis, hemostasis, and inflammation. Here we designed a physiological flow (PF) model based on short-term temporal changes in blood flow observed *in vivo* and compared it to static culture and steady flow (SF) at a fixed pulse frequency of 1.3 Hz. Results show significant changes in gene regulation as a function of temporally variable flow, indicating a reduced wound phenotype more representative of quiescence. EC cultured under PF exhibited significantly higher endothelial nitric oxide synthase (eNOS) activity (PF: 176.0±11.9 nmol/10^5^ EC; SF: 115.0±12.5 nmol/10^5^ EC, p = 0.002) and lower TNF-a-induced HL-60 leukocyte adhesion (PF: 37±6 HL-60 cells/mm^2^; SF: 111±18 HL-60/mm^2^, p = 0.003) than cells cultured under SF which is consistent with a more quiescent anti-inflammatory and anti-thrombotic phenotype. *In vitro* models have become increasingly adept at mimicking natural physiology and in doing so have clarified the importance of both chemical and physical cues that drive cell function. These data illustrate that the variability in metabolic demand and subsequent changes in perfusion resulting in constantly variable shear stress plays a key role in EC function that has not previously been described.

## Introduction

Vascular endothelial cells (EC) line the interior surface of blood vessels, providing a non-thrombogenic and selectively permeable barrier to circulating cells and macromolecules. EC are directly exposed to hemodynamic shear stress (SS), the frictional force applied by blood flow, and this stimulus is a principal mediator of EC phenotype.[1], [2] Acute changes in blood flow patterns, which occur in response to variations in cardiac output/downstream metabolic demand, also change the patterns of SS applied, thereby eliciting phenotypic adaptations (*e.g.* changes in gene transcription/protein expression) in EC.

It is has previously been demonstrated using *in vitro* SS-generating culture systems that EC behave significantly differently under SS than they do under static conditions. Applied SS causes changes in gene transcription (up/downregulation) as well as protein expression/function.[1], [2] Short-term adaptive changes to acute increases in SS (i.e. physiological increases in blood flow) include morphological reorientation of the cytoskeleton[3], [4] and intracellular protein localization[5], and stimulation of enzymatic activity.[6], [7] SS also stimulates metabolic production of endothelial-derived paracrine factors that regulate the physiology of both cells of the vascular wall (e.g. smooth muscle cells/fibroblasts) as well as those in the circulation (e.g. platelets, leukocytes, and stem cells).[1], [7]

*In vivo*, the SS waveform to which the endothelium is exposed is dependent on blood flow conditions that vary by cardiovascular load, downstream metabolic demand, and local vascular geometry. As a result, EC phenotype is spatially heterogeneous throughout the vasculature.[8] Supporting clinical evidence exists in the focal development of atherosclerotic lesions in areas of the vasculature that experience disturbed (e.g. oscillatory or reversing) blood flow patterns, which have been linked to endothelial dysfunction.[9]–[13] The focal development of cardiovascular disease states in areas of the vascular wall exposed to disturbed blood flow underscores the sensitivity of the endothelium to variations in applied SS patterns.

To better understand the molecular signals underlying these phenotypic discrepancies, a number of useful computational models have been developed to recreate *in vitro* the atheroprotective/atherogenic SS profiles to which EC are exposed in various locations throughout vascular wall.[14]–[16] The adaptation of EC to deleterious SS patterns, such as shear gradients or flow oscillation, has been characterized by increased expression of atherogenic transcription factors, such as NF-kB, leading to a sustained pro-inflammatory state.[14], [15], [17]–[19] In contrast, exposure of EC to unidirectional, laminar flow downregulates inflammatory cell adhesion molecules and cytokines, and increases production of relaxing factors such as NO that inhibit cell adhesion, migration, and proliferation.[13], [16], [18]

An equally important consideration in the regulation of EC phenotype by hemodynamic SS is the dynamic nature of blood flow rate with respect to temporal demand. *In vivo*, homeostatic changes in cardiac output vary blood flow and pulse frequency to meet metabolic demand (e.g. during exercise), and so SS patterns at the blood-vascular endothelium interface is dynamic and in a constant state of change.[20] Local hemodynamic shear patterns, whether atherogenic or atheroprotective, are therefore not fixed mechanical stimuli but highly dynamic in terms of magnitude, amplitude, and duration.

Here we tested the hypothesis that temporal changes in applied SS patterns have an important influence on EC phenotypic expression. In this study, we designed an *in vitro* model of physiological flow primarily intended to mechanically stimulate EC across a variable range of SS, rather than a fixed or steady-state stimulus, which has been common in most model systems. Results highlight the significant phenotypic differences between primary human EC cultured under temporally modulated and steady pulsatile flow *in vitro* as relevant to their critical roles in thrombosis, hemostasis, and inflammation.

## Materials and Methods

### Ethics statement

Experiments involved de-identified human tissue samples were approved according to the Institutional Review Board-01 (Gainesville, FL; IRB approval #64-2010). Because tissue samples were indirectly obtained, and de-identified prior to collection, informed consent was not deemed necessary by the Institutional Review Board.

### Endothelial cell isolation and expansion

Human umbilical cords were obtained from Labor & Delivery at Shands Hospital at the University of Florida (Gainesville, FL) and processed within 12 hours of delivery (IRB approval #64-2010). Human umbilical vein endothelial cells (EC) were isolated using collagenase perfusion as previously described.[21] Primary EC from three donors were pooled to reduce phenotypic variance. EC were maintained with VascuLife VEGF culture medium (LifeLine Cell Technologies) supplemented with 100 U/mL penicillin/streptomycin (HyClone), passaged every 2–3 days, and used experimentally between passages 2–4.

### HL-60 cell culture

HL-60 promyelocytic leukemia cells (ATCC) transduced with a green fluorescent protein-expressing lentiviral vector were generously provided by Dr. Christopher Cogle (University of Florida Department of Medicine, Gainesville, FL). They were maintained at concentrations between 5×10^5^ and 2×10^6^ cells/mL in Dulbecco’s Modified Eagle Medium supplemented with 20% FBS and 100 U/mL penicillin/streptomycin. Media was replenished every 2 days.

### Endothelial cell perfusion culture

**Figure 1:**
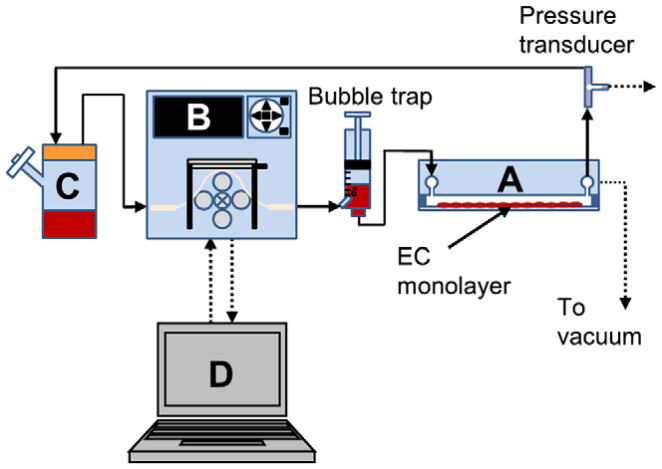
Parallel plate culture system. Endothelial cell monolayers were grown to confluence on glass coverslips, then affixed to parallel plate flow chambers (A) using a vacuum pump. Peristaltic pumps (B) are used to impose pulsatile flow of culture media (C) over the endothelial monolayers. The rotational speed of the pumps is controlled by an external computer (D) via RS-232 linkage.

EC were seeded onto glass cover slips and allowed to grow to confluence over 48 hours before initiating flow. Monolayers were affixed to parallel plate flow chambers using a vacuum pump and connected to a media reservoir fitted with a 0.22 micron air filter for gas exchange. The entire system was placed in a dehumidified incubator maintained at 37°C and 5% CO_2_. Masterflex Linkable Instrument Control Software V3.1 was used to control digital peristaltic pump drives (Masterflex) that generated pulsatile flow of media through each chamber (see Fig. 1). The mean wall SS (T) to which EC monolayers were exposed was calculated according to the Hagen-Poiseuille equation (assuming steady flow):
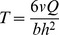



where v is media viscosity, Q is mean volumetric flow rate, and b and h are the base width and channel height, respectively. Mean volumetric flow was measured empirically, and system pressure was monitored using TruWave pressure transducers with Sonometrics SonoLAB digital acquisition system.

### qPCR

After 24 hours, EC were collected and mRNA was purified using the RNAqueous-4PCR Kit (Ambion) according to instructions. mRNA was then normalized to an equivalent amount (400 ng per sample) and reverse-transcribed to cDNA using the RT^2^ First Strand kit (SABiosciences) according to instructions. cDNA was combined with RT^2^ SYBR Green qPCR Master Mix and loaded onto human EC biology PCR arrays (SABiosciences). Amplification was performed at 95°C for 10 minutes, followed by 40 cycles of (95°C for 15 seconds and 60°C for 60 seconds). The comparative C_T_ method was used to quantify gene expression relative to the housekeeping genes *GAPDH*, *RPL13A*, *B2M*, *ACTB*, and *HRP*; EC cultured under static conditions were used as calibrating samples. Gene expression was reported as fold changes relative to calibrating samples; downregulated genes were reported inversely as negative fold changes.

### Immunocytochemistry

After conditioning, EC were gently rinsed in PBS, formalin-fixed, and co-stained using rhodamine phalloidin/DAPI (Invitrogen). Images were obtained with a Zeiss AxioImager M2 fluorescence microscope using a Zeiss AxioCam Hrm Rev 3 digital camera operated by AxioVision 4.8.

### Nitrate/nitrite quantification

Total nitrate/nitrite content (stable salt derivatives of nitric oxide) in conditioned media was quantified using the Nitrate/Nitrite Fluorometric Assay Kit (Cayman Chemical). 4–6 replicate samples were analyzed per flow condition, and results were normalized by cell number.

### HL-60 cell adhesion assay

Monolayers were activated during the final four hours of flow with 1 unit (0.16 ng/mL) recombinant human TNF-a (Thermo Scientific). At hour 24, cover slips were removed from flow circuits, rinsed in media, and incubated with a bolus (10^6^ cells/mL) of GFP-expressing HL-60 cells within Petri dishes for 10 minutes. Monolayers were rinsed 3 times in PBS and stained as described above. The number of adherent HL-60 cells were quantified in 15 specified locations throughout the flow field for each condition (n = 6).

### Statistical analysis

Results are presented as mean±SEM. One-way ANOVA followed by post-hoc Tukey-Kramer HSD analyses (with the significance level set at 0.05) were conducted to compare gene expression by experimental group or NO production by group or time. Alternatively, when equal variances could not be assumed, NO production by group was compared using Tamhane’s T2 test. Student’s t-test (significance set at 0.05) was used to compare HL-60 cell adhesion.

## Results

**Figure 2:**
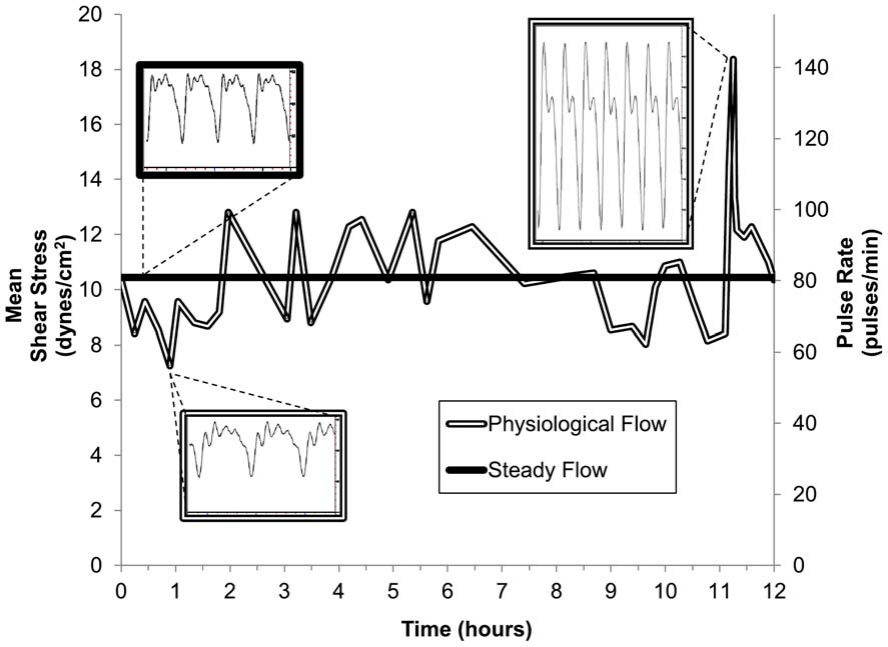
Physiologically modeled perfusion culture. A 12–hour perfusion regime (hollow line) was developed by programming a series of real-time recordings in heart rate obtained in a healthy male subject into peristaltic pumps as ramp changes. For comparison, the average shear stress magnitude calculated over the entire 12–hour cycle (10.3 dynes/cm^2^ at 80 pulses/min) was programmed as steady pulsatile flow (dark line). Mean wall shear stress and pulse frequency experienced by the endothelial cells within parallel plate flow chamber over time are shown on the left and right axes, respectively. Insets show pressure waveforms experienced at various time points during steady (dark border) or physiological flow (hollow border).

We developed a flow regime that mimics constant physiological variability associated with cardiac output to mechanically stimulate endothelial monolayers across a physiological range of arterial shear stress (SS). Real-time recordings of human heart rate in a healthy male subject were obtained over a single 12-hour period and programmed into computer-driven peristaltic pump drives so that rotational speed corresponded to the observed changes in cardiac output. The calculated mean wall SS within parallel plate flow chambers downstream of the pumps (see Fig. 2) ranged from a minimum 7.2 dynes/cm^2^ at 56 pulses/min to a maximum 18.4 dynes/cm^2^ at 142 pulses/min. For comparison, steady pulsatile flow was applied at a fixed rate (10.3 dynes/cm^2^ at 80 pulses/min); this was the calculated mean flow rate/pulse frequency from the modeled flow program averaged over the entire 12 hour cycle. Thus, both flow regimes applied the same total magnitude of SS, albeit at different rates.

### Physiologically modeled flow induces morphological adaptation in endothelial cells

**Figure 3:**
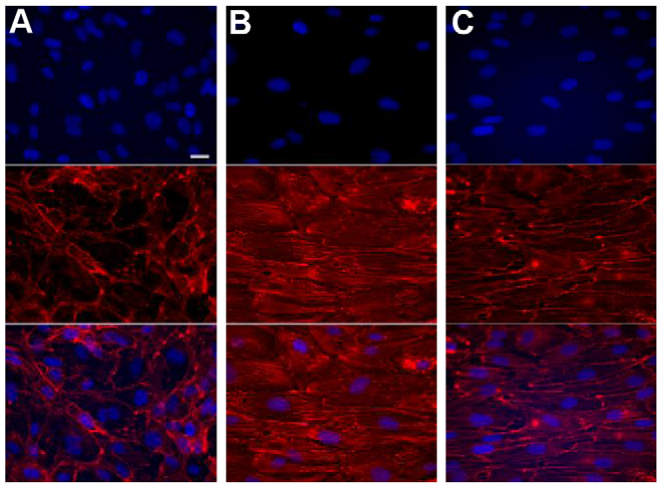
Cytoskeletal morphology of flow-conditioned endothelial cells. Endothelial cell monolayers were grown to confluence and cultured under static conditions (A), steady flow (B), or physiological flow (C) for 24 hours. Monolayers were subsequently fixed and co-stained with rhodamine phalloidin (middle row) and DAPI (top row) in order to visualize f-actin and cell nuclei, respectively. Shown are representative images (40x) from each condition. Applying shear stress resulted in cytoskeletal alignment of endothelial cells in the flow direction (horizontally left to right). Scale bar: 20 microns.

Primary human endothelial cell (EC) monolayers were grown to confluence on glass cover slips and cultured under one of three experimental conditions for 24 hours: static culture, steady flow (SF), or physiological flow (PF). For PF, two cycles were applied back-to-back to normalize the 24 hour perfusion culture period. EC maintained under static culture conditions grew to a higher density than those cultured under flow, with no global cytoskeletal organization (see Fig. 3A). As expected, culturing monolayers under laminar flow (SF or PF) induced alignment of cytoskeletal F-actin fibers in the flow direction (see Fig. 3B, C). No significant global differences in cytoskeletal morphology were apparent between EC cultured under SF or PF after 24 hours, indicating similar adaptation to both shear regimes.

### Physiological flow induces cardio-protective gene expression in endothelial cells

**Figure 4:**
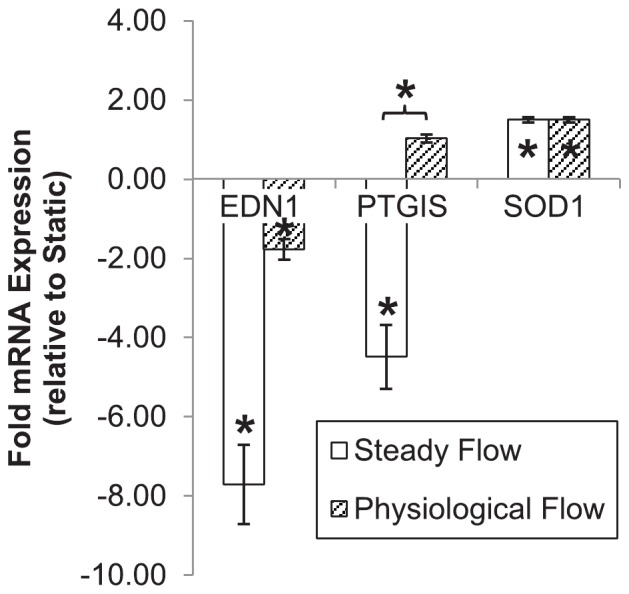
Cardio-protective gene expression in flow-conditioned endothelial cells. Shown is fold mRNA expression (with respect to static-cultured EC) of genes that promote or inhibit cardiovascular disease progression. Results are presented as mean±SEM. An embedded asterisk indicates a significant difference with respect to static controls; an asterisk over a bracket indicates a significant difference between flow groups. Abbreviations: *EDN1*: endothelin-1; *PTGIS*: prostacyclin synthase; *SOD1*: superoxide dismutase-1.

After 24 hours of conditioning under static culture, SF, or PF, EC mRNA was extracted, reverse-transcribed to cDNA, and amplified using qPCR. We assessed expression of several EC genes which, due to their importance in vascular physiology, are standard clinical diagnostic markers of cardiovascular health or disease (see Fig. 4). Maintaining EC under 24 hours of either SF or PF resulted in upregulation of superoxide dismutase-1 (*SOD-1*), an enzyme protective against oxidative stress (SF: 1.493±0.064-fold, p = 0.001; PF: 1.495±0.065-fold, p = 0.001). Furthermore, the vasopressive gene endothelin-1 (*ET-1*), which causes smooth muscle cell contraction and vasoconstriction and is implicated in atherosclerosis[22], was downregulated by both types of flow (SF: −7.710±0.999-fold, p = 0.002; MF: −1.775±0.258-fold, p = 0.048), with no significant difference between flow groups (p = 0.055). EC expression of prostacyclin synthase, a potent vasodilator and inhibitor of platelet aggregation, and characteristic marker of endothelial quiescence, was dependent on the flow regime imposed. Interestingly, *PTGIS* was dramatically downregulated by SF (−4.489±0.809-fold, p = 0.004), but unaffected by PF (1.032±0.104-fold, p = 0.171).

### Physiological flow does not significantly modulate expression of coagulation/fibrinolysis genes in endothelial cells

**Figure 5:**
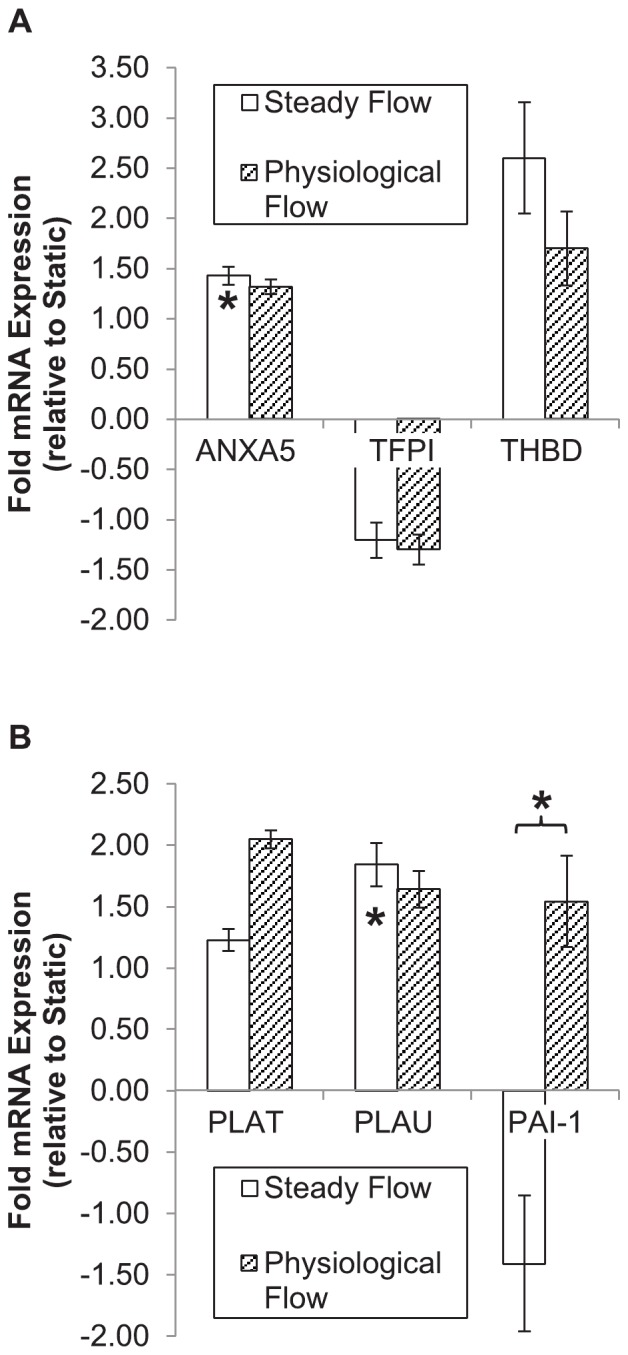
Coagulation & fibrinolysis-associated gene expression in flow-conditioned endothelial cells. Shown is fold mRNA expression (with respect to static-cultured EC) of genes associated with hemostasis (A) and fibrinolysis (B). Results are presented as mean±SEM. An embedded asterisk indicates a significant difference with respect to static controls; an asterisk over a bracket indicates a significant difference between flow groups. Abbreviations: *ANXA5*: annexin V; *TFPI*: tissue factor pathway inhibitor; *THBD*: thrombomodulin; *PLAT*: tissue plasminogen activator; *PLAU*: urokinase plasminogen activator; *PAI-1*: plasminogen activator inhibitor-1.

Distinct expression trends were found in EC genes associated with coagulation and fibrinolysis (see Fig. 5) between static culture and SF. Annexin A5 (*ANXA5*), a plasma protein with anticoagulant properties, was upregulated by SF (1.429±0.089-fold, p = 0.015), but not significantly affected by PF (1.317±0.073-fold, p = 0.062). Anti-clotting proteins tissue factor pathway inhibitor (*TFPI*) and thrombomodulin (*THBD*) did not significantly vary by experimental group. Fibrinolytic enzyme urokinase plasminogen activator (*PLAU*) was upregulated by SF alone (1.844±0.223-fold, p = 0.031), and plasminogen activator inhibitor (*PAI-1*) expression was significantly higher in cells conditioned under PF than SF (1.541±0.227-fold vs. −1.411±0.145-fold, p = 0.034), though neither deviated significantly from static culture. Overall, SF lowered expression of procoagulant genes and increased expression of fibrinolytic proteins, and while PF evoked similar trends, none significantly deviated from static culture.

### Physiological flow induces higher endothelial cell expression of chemotactic factors than steady flow

**Figure 6:**
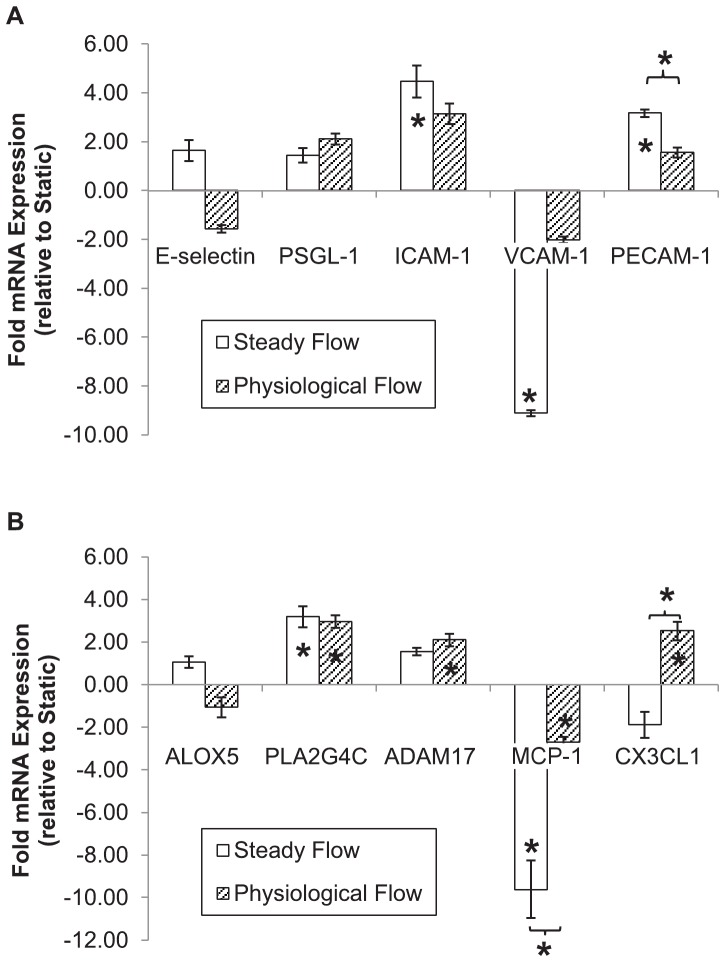
Inflammation-associated gene expression in flow-conditioned endothelial cells. Shown is fold mRNA expression (with respect to static-cultured endothelial cells) of cell adhesion molecules (A) and genes with roles in recruitment of inflammatory cells (B). Results are presented as mean±SEM. An embedded asterisk indicates a significant difference with respect to static controls; an asterisk over a bracket indicates a significant difference between flow groups. Abbreviations: *PSGL-1*: P-selectin glycoprotein ligand-1; *ICAM-1*: intercellular adhesion molecule-1; *VCAM-1*: vascular cell adhesion molecule-1; *PECAM-1*: platelet-endothelial cell adhesion molecule-1; *ALOX5*: arachidonate 5-lipoxygenase; *PLA2G4C*: cytosolic phospholipase A2 gamma; *ADAM17*: ADAM metallopeptidase domain 17; *MCP-1*: monocyte chemoattractant protein-1; *CX3CL1*: fractalkine.

EC expression of genes associated with inflammation varied greatly depending on flow conditions (see Fig. 6A,B). Culture under SF resulted in upregulation of the cell adhesion molecules ICAM-1 (4.467±0.660-fold, p = 0.006) and PECAM-1 (3.180±0.152-fold, p = 0.000), and downregulation of VCAM-1 (−9.104±0.116-fold, p = 0.006) compared with static culture. No PF-conditioned EC adhesion molecules significantly varied in expression relative to static controls, though PECAM-1 expression was significantly lower (1.566±0.196-fold, p = 0.000) when compared to steady flow.

Expression of monocyte chemoattractant protein-1 (*MCP-1*) was significantly downregulated under either flow condition, though significantly more so in EC conditioned under SF (−9.620±1.352-fold) than PF (−2.715±0.246-fold, p = 0.049). Fractalkine (*CX3CL1*) was upregulated in PF-conditioned cells only (2.522±0.437-fold, p = 0.028), and was significantly higher than in cells cultured under SF (−1.882±0.611-fold, p = 0.004). *ADAM17*, an enzyme that mediates TNF-a shedding from the cell membrane, was upregulated by PF only (2.107±0.297-fold, p = 0.035). However, cytosolic phospholipase A2 gamma (*PLA2G4C*) expression was upregulated irrespective of flow (SF: 3.190±0.501, p = 0.015, PF: 2.966+0.293, p = 0.036).

### Physiological flow induces sustained endothelial nitric oxide synthase activity

**Figure 7:**
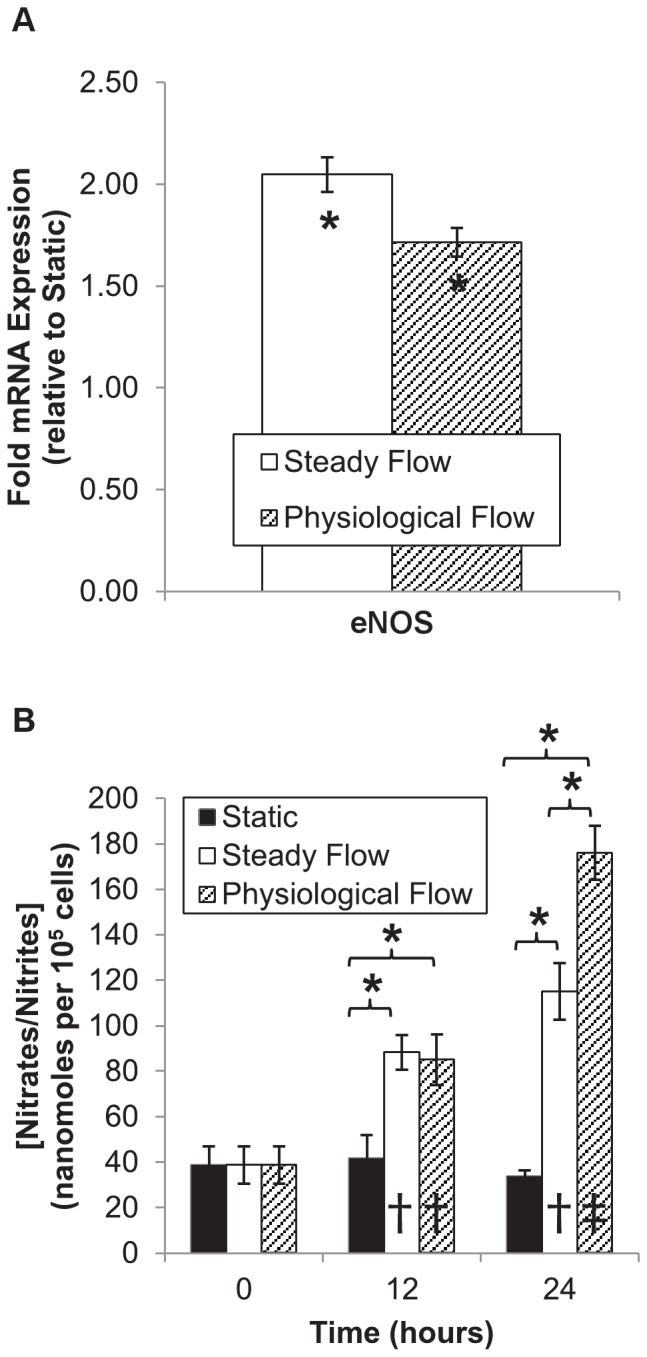
eNOS function in flow-conditioned endothelial cells. (A): eNOS mRNA expression (presented as mean±S.E.M.) was upregulated under either perfusion condition, but no statistical difference between flow groups was observed. (B): After 0, 12, or 24 hours of conditioning, media was collected and samples analyzed using a fluorometric assay. Total NO byproduct accumulation was normalized by the mean cell count at the end of each period. Results are displayed as mean±SEM (n = 4–6). Asterisks denote significant differences in individual means between groups at each time point. Dagger (†) denotes a significant difference in mean with respect to t = 0 hours. Double dagger (‡) denotes significance with respect to t = 0 and t = 12 hours.

Endothelial nitric oxide synthase (eNOS) converts L-arginine to L-citrulline and nitric oxide, the latter of which is an important inhibitor of leukocyte adhesion, platelet aggregation, and smooth muscle proliferation. This enzyme is constitutively expressed, yet highly regulated in EC.[23], [24] Nitric oxide is an important signaling molecule in vascular physiology, as its inhibition of leukocyte adhesion and platelet aggregation facilitates laminar, uninterrupted blood flow. EC expression of *eNOS* mRNA was significantly upregulated after 24 hours of flow conditioning (SF: 2.049±0.086-fold, p = 0.000; PF: 1.715±0.069-fold, p = 0.003, see Fig. 7A). However, there was no significant difference in *eNOS* gene expression between the two flow groups (p = 0.076).

eNOS enzymatic activity was assessed by quantifying the total amount of nitrates/nitrites, stable salt derivatives of nitric oxide, in conditioned culture media after 0, 12, or 24 hours of culture and normalized by EC number (see Fig. 7B). Under static culture conditions, NO content did not significantly vary by culture time: (0 hours: 38.745±8.144 nmol/10^5^ EC, 12 hours: 41.552±10.308 nmol/10^5^ EC, 24 hours: 33.680±2.614 nmol/10^5^ EC, p = 0.689). After 12 hours of perfusion culture, significant increases in NO byproduct accumulation were observed (SF: 88.308±7.608 nmol/10^5^ EC, p = 0.025; MF: 84.993±11.233 nmol/10^5^ EC, p = 0.030), with no significant difference in means observed between flow groups (p = 0.969).

However, noteworthy deviations in NO output occurred between flow regimes during the last 12 hours of perfusion. No significant increase in nitrates/nitrites was observed between 12 and 24 hours of SF (12 hours: 88.308±7.608 nmol/10^5^ EC, 24 hours: 115.039±12.460 nmol/10^5^ EC, p = 0.186). PF, however, induced significant temporal increases in NO output between 12 (84.993±11.233 nanomoles/10^5^ EC) and 24 hours (176.0±11.860 nanomoles/10^5^ EC, p = 0.000) of conditioning. After 24 hours, the highest nitrate/nitrite concentrations were measured in media from EC cultured under PF, which was significantly higher than that from EC cultured under SF (p = 0.002).

### Nitric oxide production by endothelial cells is significantly higher with programmed shear changes than constant-frequency flow

For added clarity, two additional flow groups were included to profile nitric oxide production under physiologically dynamic conditions. First, another steady flow group (SF-160) was included in which the flow was applied at twice the rate of the SF (160 pulses/min, 20.6 dynes/cm^2^). Similar trends were observed under SF-160 as normal SF; no significant differences in nitrates were observed between the two groups (see Fig. S1). Second, the physiological flow model was applied in reverse chronological order. Reversed physiological flow (rPF) induced a significant increase in nitrates (relative to static culture, SF-80, and SF-160) after 6 hours that was maintained through 24 hours of culture (see Fig. S1). However, no significant differences in nitrates/nitrites were observed between PF and RF at any of the time points assessed.

### Physiological flow enhances endothelial cell resistance to activation-induced leukocyte adhesion

**Figure 8:**
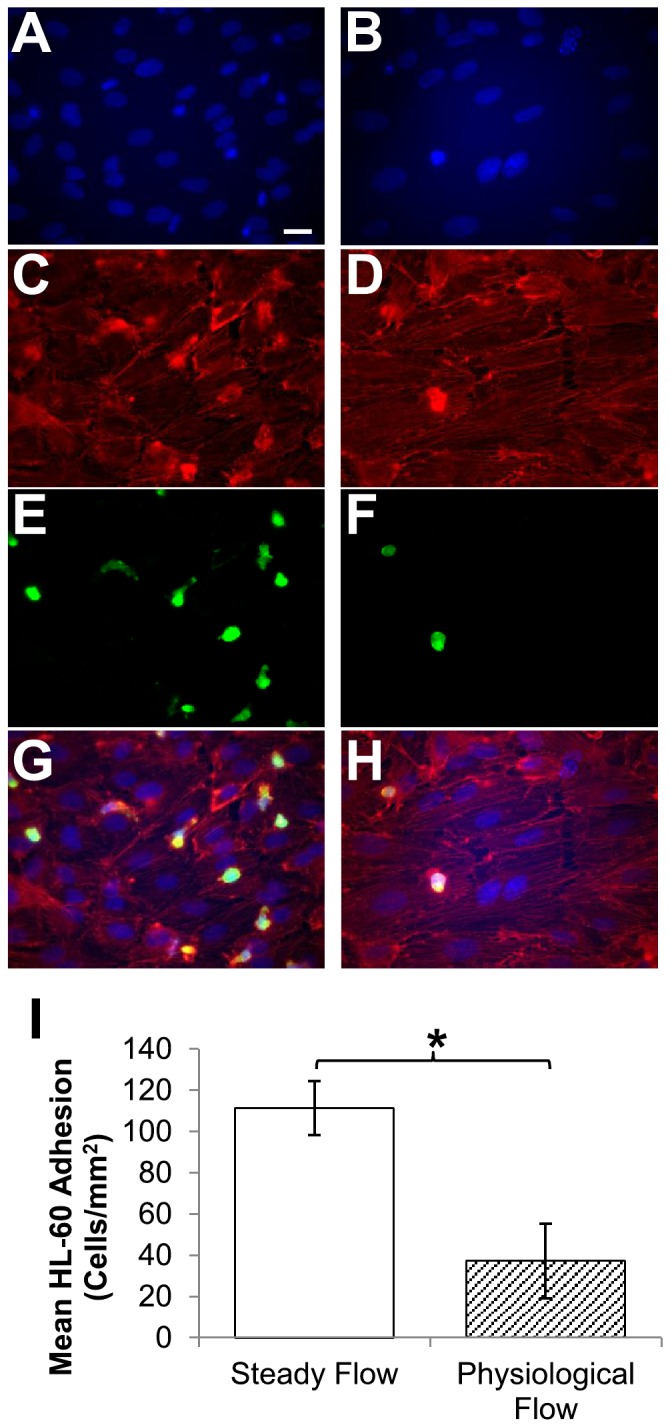
HL-60 cell adhesion to flow-conditioned endothelial cells. Endothelial cell monolayers were grown to confluence and cultured under steady flow (A,C,E,G) or physiological flow (B,D,F,H) for 24 hours. During the last four hours, endothelial cells were activated with 1 U TNF-a to stimulate adhesion molecule expression. At hour 24, monolayers were removed from flow chambers and incubated for 10 minutes with a bolus of GFP+ HL-60 cells (1000 cells/mm^2^) and stained as described (A,B: DAPI; C,D: F-actin; E,F: GFP+ HL-60 cells; G,H: overlay). Shown are representative images (40x) from each condition. Scale bar: 20 microns. (I): HL-60 cell adhesion in 15 predetermined locations per monolayer was quantified. Results are displayed as mean±SEM (n = 5–6). Asterisk denotes significant difference in means between groups as determined by Student’s t-test.

Given the dramatic increase in NO production noted in PF-conditioned EC compared with steady flow, we next performed a functional assay to characterize the extent to which temporal changes in shear influenced EC adhesiveness for leukocytes. After four hours of activation with TNF-a, flow-conditioned monolayers were transferred to Petri dishes and incubated with a bolus of promyelocytic GFP^+^ HL-60 cells (see Fig. 8). Conditioning EC under PF significantly reduced HL-60 leukocyte attachment compared to conditioning under SF (PF: 37±6 HL-60 cells/mm^2^; SF: 111±18 HL-60/mm^2^, p = 0.003).

## Discussion

Vascular endothelial cell (EC) phenotype is a function of multiple physiological factors, of which appropriate shear stimulation is critical for maintaining EC in a quiescent, non-thrombogenic state. Defining how hemodynamic shear patterns mediate EC function, as well as pathological dysfunction, is critical to our understanding of vascular physiology.[9], [11], [25], [26]

*In vitro* systems designed to expose EC to controlled levels of fluid shear stress (SS) have greatly advanced our understanding of the molecular mechanisms underlying the transduction of hemodynamic shear stress into biological signals.[2], [27] It is now recognized that specific details of the shear waveform have important implications for EC adaptation/functionality. For example, EC morphology, gene expression, enzymatic activity have all been shown to vary depending on whether flow is applied steadily or with pulsation, as occurs *in vivo*.[15], [28], [29] It has become increasingly clear that recapitulating the complex characteristics of physiological blood flow is critical to the utility of *in vitro* model systems.

A significant gap lies between the majority of *in vitro* perfusion culture systems and physiological blood flow patterns seen *in vivo*.[25] Although experimental flow models accurately recreate the shear waveforms experienced at specific locations on the vascular wall[14]–[16], the waveform is most often applied cyclically, without modulation of frequency or amplitude for the duration of the experiment. The temporal variations in blood flow rate that continuously occur *in vivo* are not taken into account. Given that EC phenotype has been shown to be a function of pulse frequency[30], SS magnitude[31], [32], and pulse amplitude[33], all of which vary *in vivo* throughout the course of the day, blood flow dynamics warrant consideration in these systems.

Here we tested the hypothesis that temporal variability in applied SS would induce changes to EC phenotype as relevant to their critical roles in circulatory/vascular biology. The physiological flow (PF) model in this study was developed to simulate physiological changes in pulse rate and volumetric flow rate as they occur *in vivo* over the course of a single day. The model encompassed a range of SS commonly found in small diameter arteries.[34]–[36] Steady flow with an equivalent time-averaged SS and fixed pulse frequency was used to represent the conditions used in current systems.

Transcriptional profiling of EC exposed to steady pulse frequencies versus physiologically modeled flow demonstrated significant differences in gene expression. Prostacyclin synthase, the enzyme responsible for production of a potent vasodilator/inhibitor of platelet aggregation, was significantly downregulated in cells conditioned under steady flow, while no change was induced by physiological flow. The leukocyte adhesion molecule/chemokine fractalkine was upregulated under physiological flow alone. In some cases, gene expression trends (relative to static-cultured cells) were statistically similar regardless of flow regime (protective enzymes *eNOS, SOD-1* were upregulated; leukocyte chemokine *MCP-1* was downregulated). Elucidating the various signaling mechanisms involved in gene transcription regulation by SS is an area of intense interest. The transcription factor KLF-2, for example, has been found to play a role in eliciting the atheroprotective effects of laminar SS in EC, both through induction of eNOS expression as well as inhibition of agonist-induced expression of E-selectin and VCAM-1.[18] Boon *et al* have published a review on several known SS-responsive transcription factors[37], and the role of these factors in the presently observed results will be the topic of future investigations. Because only endpoint gene expression analysis was performed, temporal trends were not observed and comparisons between the PF model and the steady control at 24 hours alone limits the number of conclusions that may be drawn. Taken collectively, however, the data show a more quiescent EC transcriptome elicited by both flow regimes compared to statically cultured cells.

Shear stimulation of EC has important implications for metabolic production of nitric oxide (NO), an important inhibitor of platelet aggregation[38], leukocyte adhesion[39], and smooth muscle cell mitogenesis[40]. Diminished NO bioavailability is a hallmark of endothelial dysfunction, which is a prevalent symptom in a wide variety of cardiovascular disorders, including thrombosis[41], intimal hyperplasia[42], pulmonary hypertension[43], and atherosclerosis.[44] In the present study, notable increases in nitric oxide (NO) production were observed when EC were exposed to PF, a regime characterized by temporal increases (and decreases) in SS magnitude/pulse frequency. In agreement with previous studies, we found that application of SS caused upregulation of eNOS gene expression.[28], [45] Though eNOS expression between cells exposed to each flow regime did not statistically vary, the metabolic activity of the enzyme changed dramatically. While endothelial NO production tapered off after 12 hours of steady flow, physiological flow induced dramatic increases in NO synthesis between 12 and 24 hours.

Previous research has shown that endothelial NO production is dependent on not only the overall magnitude but also the rate at which shear stimulation is applied. [6], [28], [46] While rapid increases in SS induce a transient G protein coupled receptor-dependent burst in NO production, steady flow results in a state of sustained NO production.[47] We postulate that the tapering off in NO output is due to physiological adaptation of EC to the steady-state conditions associated with fixed-pulse flow. SS modulates eNOS activity in EC through a variety of mechanisms: calmodulin binding, phosphorylation state of certain residues, and association with membrane-bound proteins such as PECAM-1.[23], [48], [49] In particular, acute SS increases have been shown to elevate eNOS activity through phosphorylation of Ser1177 residue by PI-3K.[50] The rapid increases in shear within the physiological flow regime likely stimulated periodic bursts in NO production that were not induced under steady flow. The significantly lowered leukocyte adhesiveness in physiological flow-conditioned EC observed correlates with higher NO production, as well as significantly lower expression of PECAM-1 when compared to steady flow.[51]

The present evidence suggests EC quiescence can be induced by dynamic stimulation across a physiological range of arterial shear stresses. It is therefore plausible to consider that temporal alterations in mechanical stimulation may affect functionality of other cell types as well. Sydeain *et al* previously demonstrated increased ERK signaling and greater collagen synthesis by human dermal fibroblasts cultured in a cyclic distension bioreactor when incremental increases in stretch were applied, rather than fixed distension.[52] Though disparities exist between the two model systems, both show evidence that the response of cells to mechanical stimulation is dependent on the overall magnitude/frequency of the stimulus. Application of variable mechanical stimuli to cells cultured in other bioreactor systems that may influence regeneration of blood vessels, tendon, bone, cartilage, or other tissues *in vitro*.

In conclusion, we have demonstrated the inherent sensitivity of critical EC functions to temporal changes in applied SS. The steady state flow applied in current culture systems, while useful for close examination of molecular signaling events, may not accurately represent the physiological hemodynamics to which EC are exposed *in vivo*. These investigations show a clear EC phenotype modulation toward a quiescent arterial state and improved functionality in a simulated wound environment. Understanding the conditions that regulate EC function and having the capacity to model these appropriately *in vitro* has significant implications for treatment of clinical pathologies associated with endothelial dysfunction.

## Supporting Information

Figure S1 **Nitric oxide profiling of endothelial cells exposed to additional flow conditions.** Endothelial cells were cultured under the conditions previously described (static, steady flow [SF-80], or physiological flow [PF]) as well as two additional control groups. *SF-160:* The rotational speed of the pump was doubled, so that the pulse frequency (160 pulses/min) and mean shear stress (20.6 dynes/cm^2^) applied were twice that of every other flow group. *RF:* Additionally, the physiological flow cycle (PF; see Fig. 2) was applied chronologically in reverse. Media was collected and samples analyzed using a fluorometric assay. Total NO byproduct accumulation was normalized by the mean cell count at the end of each period. Results are displayed as mean±SEM (n = 4). Asterisks denote significant differences in individual means between groups at each time point.
(TIF)
