# Supplementary figures and images for: Adaptation of Endothelial Cells to Physiologically-Modeled, Variable Shear Stress
Source: PLoS One. 2013 Feb 14;8(2):e57004. doi: 10.1371/journal.pone.0057004 (PMC3573044; doi:10.1371/journal.pone.0057004)

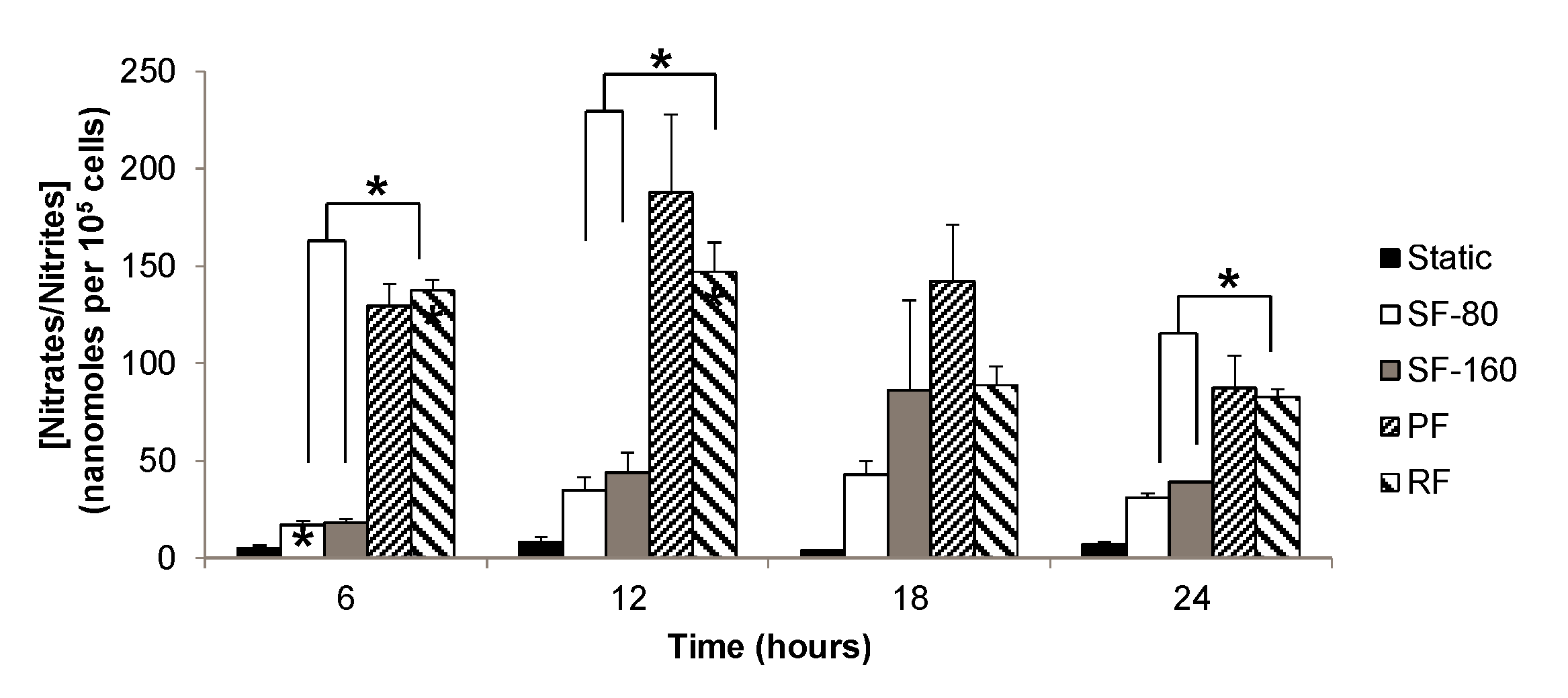

Supplement: Figure S1 — Nitric oxide profiling of endothelial cells exposed to additional flow conditions. Endothelial cells were cultured under the conditions previously described (static, steady flow [SF-80], or physiological flow [PF]) as well as two additional control groups. SF-160: The rotational speed of the pump was doubled, so that the pulse frequency (160 pulses/min) and mean shear stress (20.6 dynes/cm2) applied were twice that of every other flow group. RF: Additionally, the physiological flow cycle (PF; see Fig. 2) was applied chronologically in reverse. Media was collected and samples analyzed using a fluorometric assay. Total NO byproduct accumulation was normalized by the mean cell count at the end of each period. Results are displayed as mean±SEM (n = 4). Asterisks denote significant differences in individual means between groups at each time point. (TIF) [file pone.0057004.s001.tif]
